# Associations between fear of cancer recurrence and post-traumatic growth in patients with primary liver cancer: a latent profile analysis and mediation analysis

**DOI:** 10.3389/fpsyt.2026.1819344

**Published:** 2026-06-08

**Authors:** Qunfeng Zou, Chengfeng Xu, Yuan Liao, Yuqing Sun, Dingrong Qiu, Lijun Lin

**Affiliations:** 1 The Second Clinical Medical College, Guangzhou University of Chinese Medicine, Guangzhou, China; 2 School of Nursing, Guangzhou University of Chinese Medicine, Guangzhou, China; 3 Department of Nursing, The Second Affiliated Hospital of Guangzhou University of Chinese Medicine, Guangzhou, China

**Keywords:** fear of cancer recurrence, post-traumatic growth, primary liver cancer, self-efficacy, social support

## Abstract

**Background:**

Promoting post-traumatic growth in patients with liver cancer is of great significance for improving their quality of life and psychosocial adaptation. Previous studies have demonstrated that fear of cancer recurrence (FCR) is a risk factor that hinders post-traumatic growth (PTG). However, existing research often overlooks the heterogeneity of fear of cancer recurrence, and the pathway differences between distinct latent classes and post-traumatic growth remain unclear. Therefore, this study aims to identify the latent classes of fear of cancer recurrence in patients with primary liver cancer and to examine the mediating roles of perceived social support and self-efficacy between different classes and post-traumatic growth.

**Methods:**

The cross-sectional survey was organized between February and December 2025. Patients with primary liver cancer from a tertiary hospital in Guangzhou city were recruited through convenience sampling method. The levels of FCR, perceived social support, self-efficacy, and PTG were measured using paper-based questionnaires, and the relationships among variables were analyzed using Pearson correlation analysis, latent profile analysis, and multicategorical independent variable mediation analysis.

**Results:**

Three latent profiles of FCR were identified: low fear–psychologically well-adapted group (29.32%), high fear–social functioning concerns group (35.50%), and moderate-to-high fear–treatment concerns group (35.18%). Analysis of influencing factors showed that type of medical insurance, presence of comorbidities, and occupational status were predictors of different latent classes of fear of cancer recurrence in patients with primary liver cancer (P < 0.05). The differences were found in the total scores and subscales of perceived social support, self-efficacy, and PTG among patients with different latent FCR profiles. Mediation analysis showed that, with the low fear–psychologically well-adapted group as the reference, the relative mediation effects were significant for both the high fear–social functioning concerns group and the moderate-to-high fear–treatment concerns group.

**Conclusion:**

Fear of cancer recurrence demonstrates marked heterogeneity across individuals diagnosed with primary liver cancer. Clinicians should implement targeted interventions for patients with different FCR profiles to mitigate its negative impact and promote psychological growth by activating their social support systems and enhancing self-efficacy.

## 1 Introduction

Primary liver cancer (PLC) has become one of the major malignant tumors that seriously threaten patients’ quality of life due to its characteristics of high invasiveness, difficulty in early diagnosis, and a recurrence and metastasis risk of up to 70% within five years after surgery (1, 2). This persistent and unpredictable threat of recurrence exposes patients with liver cancer to prolonged psychological stress, among whom FCR has become the most central and common psychological distress. FCR refers to the fear, worry, or apprehension about cancer recurrence or progression (3). Previous studies have shown that approximately 60% of patients with liver cancer report clinically significant, high levels of FCR (4). High FCR not only leads to excessive vigilance, anxiety, and avoidance behaviors but also impairs fundamental cognitive functions such as future planning and social interaction, thereby severely compromising quality of life (5, 6). Existing studies on FCR have mostly focused on cancer types such as breast cancer and colorectal cancer (7, 8), while relatively little attention has been paid to the unique psychological challenges faced by patients with liver cancer, which has a higher recurrence rate and poorer prognosis. Further exploration is urgently needed.

With the development of positive psychology, researchers have increasingly focused on the positive psychological resources individuals may mobilize during a cancer-related traumatic experience. While enduring the psychological stress associated with the disease, cancer patients may also engage in positive cognitive reappraisal to alleviate distress, thereby uncovering meaning hidden within the traumatic event and achieving PTG (9). Such positive psychological changes occurring during trauma recovery are termed PTG. High levels of PTG help cancer patients effectively cope with the disease’ s impact and rebuild physical and mental well-being (10). Previous studies have predominantly reported a negative association between FCR and PTG, such that elevated FCR levels impede PTG. As a persistent source of psychological distress, FCR may indirectly hinder PTG by depleting critical psychological resources, rather than directly facilitating PTG or coexisting with it (11).

The theoretical model of life crisis and personal growth posits that a life crisis can foster positive psychological growth and development through cognitive, emotional, and behavioral self-regulation, as well as the support of external resources such as social support (12). FCR is a persistent emotional distress triggered by the crisis event of having cancer. Grounded in this model, the present study introduces perceived social support and self-efficacy as key mediating variables to examine the internal mechanisms underlying the influence of FCR on PTG. As a critical external resource, perceived social support serves multiple functions in psychological adaptation and emotion regulation—facilitating resource integration and psychological adjustment among cancer patients, thereby promoting PTG (13, 14). However, high levels of FCR are often associated with heightened sensitivity to disease-related information and negative interpretation of interpersonal support, which diminishes patients’ actual perception of and ability to utilize social support, indirectly constraining the development of positive psychology (15, 16). Furthermore, as a vital internal resource, self-efficacy refers to an individual’s belief in their capacity to manage challenges (17). Individuals with high self-efficacy are more likely to employ adaptive coping strategies and maintain a sense of hope and control in adversity, thus effectively facilitating PTG (11). Research among cancer patients has also confirmed that self-efficacy significantly buffers the adverse impact of fear of disease progression on psychological well-being (18). Conversely, persistent FCR continually undermines patients’ confidence in their coping abilities, leading to negative outcome expectations (19). Patients with low self-efficacy tend to adopt maladaptive coping styles—such as avoidance—that impede the integration of positive psychological resources and hinder PTG (20). Therefore, perceived social support and self-efficacy may serve as mediators in the relationship between FCR and PTG.

It should be clear that social support and self-efficacy are not isolated from each other but rather exhibit a sequential and interactive relationship. According to Bandura’s theory of self-efficacy (17), an individual’s sense of self-efficacy can be established or strengthened through significant external sources, such as social support. Previous research has found that social support significantly and positively predicts self-efficacy. Higher levels of perceived social support are associated with greater engagement in social interactions, enabling cancer patients to access more health information and disease management skills, thereby enhancing their self-efficacy (21). Meanwhile, social support plays a key role in cancer-related adaptation, facilitating the reconstruction of self-efficacy and thereby promoting more positive treatment outcomes for patients across the disease trajectory (22). Based on this evidence, the present study hypothesizes a sequential mediation model in which FCR influences PTG through two linked mediators: perceived social support and self-efficacy. Specifically, lower levels of FCR facilitate patients’ perception of and ability to utilize social support; enhanced perceived social support, in turn, strengthens patients’ self-efficacy; and elevated self-efficacy ultimately promotes the emergence and development of PTG.

However, previous studies constructing chain mediation models typically assume homogeneity in fear of cancer recurrence among patients—that is, the underlying mechanisms operate consistently across the group. In contrast, FCR is a multidimensional construct, and individuals may exhibit distinct FCR profiles that vary according to FCR severity and the coping strategies employed (23). Ignoring this heterogeneity will hinder the development of precise and effective interventions, potentially leading to poor intervention outcomes and wasteful use of medical resources (24). Latent Profile Analysis (LPA) is an individual-centered statistical method that identifies latent subgroups within a population based on individuals’ response patterns across observed variables (25). LPA provides a robust methodological tool for uncovering the internal structural heterogeneity of FCR and informing the development of personalized intervention strategies.

**Figure 1:**
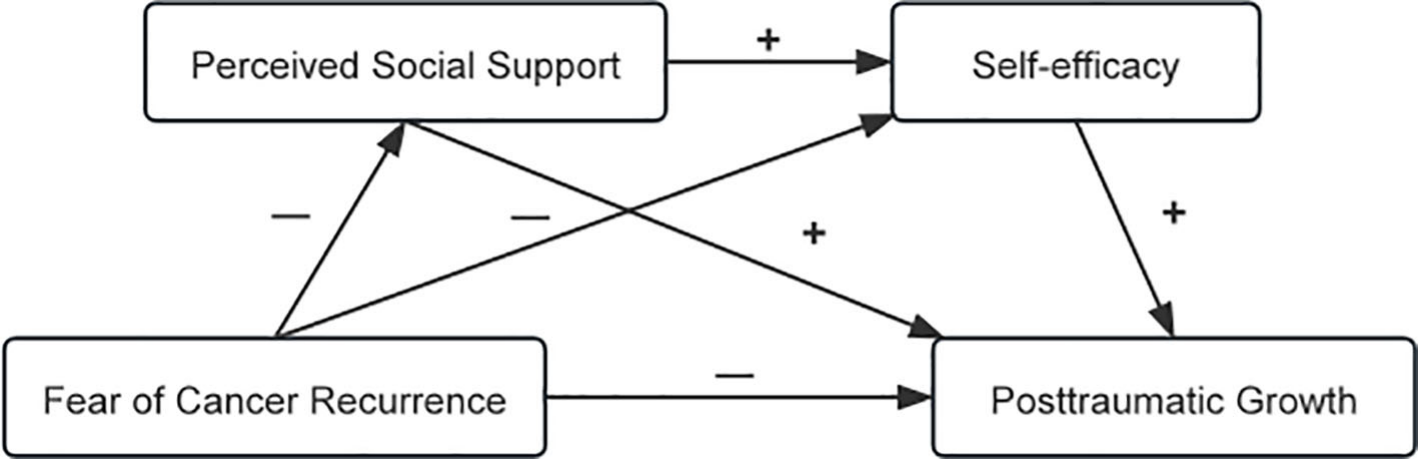
Conceptual model. The variables marked with "-" are assumed to be negatively correlated, Positive and negative arrows indicate the nature of the hypothesized associations between constructs.

In summary, grounded in the theoretical framework of life crisis and personal growth, this study employs LPA to identify potential heterogeneity in FCR among patients with primary liver cancer and to construct a moderated mediation model—with FCR subtype as the independent variable and PTG as the core outcome. To systematically examine the sequential mediating roles of perceived social support and self-efficacy in the association between distinct FCR subtypes and PTG, thereby providing an empirical basis for clinicians to identify high-risk FCR subgroups and design subtype-specific, evidence-informed psychosocial interventions. Based on the above rationale, the hypothesized conceptual model of this study (see **Figure 1**) is presented, and the research hypotheses are as follows:

H1: There are obvious heterogeneous subtypes of FCR in patients with primary liver cancer.

H2: Significant associations were found among FCR, perceived social support, self-efficacy and PTG.

H3: Perceived social support and self-efficacy play a chain mediating role between different latent profiles of FCR and PTG.

## 2 Materials and methods

### 2.1 Sampling and inclusion criteria

This study implemented a cross-sectional design and conformed to the Strengthening the Reporting of Observational Studies in Epidemiology (STROBE) reporting guidelines. Patients with primary liver cancer were recruited from a tertiary hospital in Guangdong Province, China, between February and December 2025 using a convenience sampling method. Inclusion criteria: (1)Aged ≥18; (2) met the diagnostic criteria for primary liver cancer; (3) normal cognitive function; (4) provided informed consent voluntarily. Exclusion criteria were: (1) current or prior participation in psychological intervention studies, such as mind fulness-based interventions, because interventions may systematically alter levels of FCR, perceived social support, self-efficacy, and PTG, thereby introducing unmeasured confounding; (2) patients with severe comorbid critical illnesses, such as cardiac or renal insufficiency; (3) patients diagnosed with psychiatric disorders;

The sample size for this study was estimated based on Kendall’s criterion (26). This study included 28 predictor variables. To guarantee sufficient statistical power for multivariable modeling, the minimum sample size was calculated as 5 observations per variable and further adjusted upward by 20% to account for anticipated attrition, resulting in a final target of 168 participants. Moreover, to ensure the stability and interpretability of latent profile analysis results, the sample size should exceed 200 (27). In this study, 317 questionnaires were distributed, of which 307 were completed and met all inclusion criteria, yielding an effective response rate of 96.8%. The present study received ethical approval from the Institutional Review Board of Guangdong Provincial Hospital of Chinese Medicine (Ethics Review Number: YE2025-051-01).

### 2.2 Instruments

#### 2.2.1 Demographic characteristics

This study’s instrument was designed by the research team following a literature review and consisted of two parts. The first part collected demographic information, including gender, age, education level, and type of health insurance. The second part covered clinical characteristics, including disease stage, treatment modality, time since diagnosis, disease recurrence or progression, and disease-related knowledge.

#### 2.2.2 Fear of progression questionnaire-short form

This scale was developed by Mehnert et al. (28), and the Chinese version was validated by Wu et al. and has been applied to patients with liver cancer, with an internal consistency Cronbach’s α of 0.883 (29). The scale consists of 12 items covering two domains: physical health and social/family functioning. Each item is rated on a 5-point Likert scale ranging from “never” to “always,” with scores assigned 1 to 5, respectively. A higher total score indicates a greater level of fear, and a total score ≥ 34 indicates psychological dysfunction. In this study, the scale demonstrated a Cronbach’s α of 0.803.

#### 2.2.3 Posttraumatic growth inventory-Chinese version

The original scale was developed by Tedeschi and Calhoun to assess positive psychological changes occurring in individuals following traumatic events (30). The Chinese version of the Post-Traumatic Growth Scale was translated and culturally adapted by Wang et al. and validated in Chinese cancer patients (31). It consists of 20 items covering five dimensions: appreciation of life, new possibilities, personal strength, changes in self-perception, and relating to others. Each item is scored on a 0-to-5 scale to indicate the respondent’s degree of experiencing a particular change. A higher level of posttraumatic growth is indicated by a higher entire score. In this study, the scale demonstrated a Cronbach’s α of 0.820.

#### 2.2.4 Perceived social support scale

This study utilized the research tool developed by Zimet et al. (32), which was translated into Chinese and validated by Jiang (33). PSSS comprises 12 items grouped into three aspects: family, friends, and other support. Scores on this 7-point Likert scale range from 1 to 7, with higher scores reflecting greater perceived social support. The Cronbach’s α coefficients for both the scale and each dimension ranged from 0.789 to 0.923, demonstrating strong internal consistency reliability (34). The Cronbach’s α coefficient in this study was 0.850.

#### 2.2.5 Strategies used by people to promote health

SUPPH was developed by Lev and Owen to assess participants’ self-efficacy (35). The Chinese version was translated by Qian and Yuan and validated in a Chinese cancer patient population, demonstrating good internal consistency (Cronbach’s α = 0.970) (36). This scale comprises three dimensions: positive attitude, self-stress reduction, and self-determination, with a total of 28 items. The instrument employs a 5-point Likert-type scale (1 = “no confidence”, 5 = “complete confidence”), with possible total scores ranging from 28 to 140; higher scores indicate greater self-efficacy in managing FCR. In this study, Cronbach’s α for this scale was 0.959.

### 2.3 Data quality control

The survey was conducted by two researchers who underwent standardized training. The research team clarified the study’s purpose, content, completion methods, and precautions to the participants and promptly addressed any questions raised by them. Upon completion of the survey, the researchers verified the questionnaires on-site for completeness and collected them only after confirming that there were no errors. In the data analysis process, first, the research data were entered by two people and cross-checked. Outliers were screened out using box plots or Z-score methods, and reasonable extreme values were retained. The missing rate of each variable was less than 5%, and multiple imputation was used to handle missing values. Secondly, the normality test was based on the results of data skewness and kurtosis, and the Bootstrap method was used to verify the stability of the results in key analyses. Finally, the Harman single-factor test method was used to detect common method bias.

### 2.4 Data analysis

First, descriptive statistical analyses of demographic data, disease−related information, perceived social support, self−efficacy, and post−traumatic growth in patients with primary liver cancer were performed using SPSS (Version 27.0). Normality was assessed by combining skewness and kurtosis. When the absolute value of skewness was < 1 and the absolute value of kurtosis was < 3, the data were considered approximately normally distributed. Normally distributed continuous variables were presented as mean ± standard deviation; non−normally distributed continuous variables were presented as median and interquartile range; categorical variables were presented as frequencies, constituent ratios, and rates.

Subsequently, the latent profile model was constructed using Mplus 8.3 software. The model fit indices included (1): information criteria—Akaike Information Criterion (AIC), Bayesian Information Criterion (BIC), and sample-adjusted BIC (aBIC); smaller values indicate better model fit (2); classification accuracy, assessed by entropy, which ranges from 0 to 1; values closer to 1 indicate more distinct and accurate class separation; and (3) likelihood ratio tests—including the Lo-Mendell-Rubin adjusted likelihood ratio test (LMR-LRT) and the bootstrap likelihood ratio test (BLRT). A significant result (P < 0.05) indicates that the k-class model fits significantly better than the (k − 1)-class model. The optimal model was selected based on these fit indices and substantive interpretability.

Third, one−way analysis of variance (ANOVA) and chi−square tests were used to analyze continuous and categorical variables, respectively, to compare differences in sociodemographic characteristics across subgroups. Variables that showed statistical significance in the univariate analyses were subsequently entered into a multivariate multinomial logistic regression model, deeming differences significant when P < 0.05. Subsequently, Pearson correlation analysis was performed to examine the relationships among FCR, perceived social support, self-efficacy, and post-traumatic growth.

Finally, to examine the serial mediating roles of perceived social support and self-efficacy in the relationship between different FCR profiles and post-traumatic growth (PTG), we used the PROCESS macro (Model 6) for SPSS 27.0. Because the independent variable (FCR profile) was multicategorical, we created dummy variables with the low fear–psychologically well-adapted group as the reference. Perceived social support and self-efficacy were specified as serial mediators, and PTG was specified as the outcome. To control for potential confounding, independent predictors identified from the multinomial logistic regression were included as covariates. Bias-corrected bootstrap 95% CIs (5,000 resamples) were used to test the significance of relative indirect effects. A confidence interval not containing zero indicates a statistically significant indirect effect. All continuous variables were standardized prior to analysis. Statistical significance was set at a threshold of α = 0.05.

## 3 Results

### 3.1 Participants’ characteristics

The mean age of the participants was 59.58 ± 12.21 years, with 243 patients (79.2%) being male; 202 patients (65.8%) unemployed; 264 patients (86.0%) residing in urban areas; the per capita monthly household income for 161 patients (52.4%) was below 6,000 RMB; and 161 patients (52.4%) covered by employee health insurance; 191 patients (62.2%) having a disease duration of less than one year; 222 patients (72.3%) with no history of recurrence or progression; 222 patients (72.3%) at disease stages I–II; 222 patients (72.3%) with a history of hepatitis; and more than half (51.1%) with other chronic diseases; 159 patients (50.5%) having received comprehensive treatment; and 261 patients (85.0%) having some knowledge about their disease. Additionally, regarding demographic characteristics, significant differences were observed among the profiles in employment status, place of residence, per capita monthly household income, and type of health insurance (p < 0.05). Regarding clinical characteristics, the profiles also differed significantly in the presence of comorbidities (p < 0.05). **Table 1** presents the remaining characteristics.

**Table 1:** Univariate analysis of latent profiles of fear of cancer recurrence in patients with primary liver cancer (N = 307).

Variables	Variables	C1(n=90)	C2(n=109)	C3(n=108)	*χ^2^*/*F*	*P*
gender	Male	66 (73.30%)	90 (82.60%)	87 (80.60%)	2.747^a^	0.253
	Female	24 (26.70%)	19 (17.40%)	21 (19.40%)		
Age	18~44	14 (15.60%)	16 (14.70%)	16 (14.80%)	7.472^a^	0.279
	45~59	35 (38.9%)	34 (31.20%)	24 (22.20%)		
	60~74	33 (36.7%)	47 (43.10%)	56 (51.90%)		
	75~89	8 (14.4%)	12 (11.00%)	12 (11.10%)		
Education level	Junior high school or below	54 (60.00%)	70 (64.20%)	73 (67.60%)	2.809^a^	0.590
	High school or technical secondary school	23 (25.60%)	26 (23.90%)	27 (25.00%)		
	Junior college degree or higher	13 (10.00%)	13 (11.90%)	8 (7.40%)		
Occupation status	In-service/Employed	40 (44.40%)	39 (35.80%)	26 (24.10%)	9.239^a^	0.010
	Not employed	50 (55.60%)	70 (64.20%)	82 (75.90%)		
Place of residence	urban	81 (90.00%)	84 (77.10%)	99 (91.70%)	11.301^a^	0.004
	rural	9 (10.00%)	25 (22.90%)	9 (8.30%)		
Marital status	Married	80 (88.90%)	100 (91.70%)	99 (91.70%)	0.609^a^	0.737
	Single/Divorce/Widowed	10 (11.10%)	9 (8.30%)	9 (8.30%)		
Average monthly household income	<6000 RMB	39 (43.30%)	71 (65.10%)	51 (47.20%)	11.218^a^	0.004
	≥6000 RMB	51 (56.70%)	38 (34.90%)	57 (52.80%)		
Type of health insurance	Non-employee medical insurance	28 (31.10%)	75 (68.80%)	43 (39.80%)	32.092^a^	<0.001
	Employee medical insurance	62 (68.90%)	34 (31.20%)	65 (60.20%)		
Time Since Diagnosis	<1 year	58 (64.40%)	68 (62.40%)	65 (60.20%)	1.689^a^	0.793
	1–3 years	20 (22.20%)	30 (27.50%)	27 (25.00%)		
	>3 years	12 (13.30%)	11 (10.10%)	16 (14.80%)		
Disease staging	Stage I	36 (40.00%)	47 (43.10%)	43 (39.80%)	3.828^b^	0.708
	Stage II	32 (35.60%)	35 (32.10%)	29 (26.90%)		
	Stage III	19 (21.10%)	24 (22.00%)	33 (30.60%)		
	Stage IV	3 (3.30%)	3 (2.80%)	3 (2.80%)		
Disease recurrence or progression	No	70 (77.80%)	77 (70.60%)	75 (69.40%)	1.938^a^	0.379
	Yes	20 (22.20%)	32 (29.40%)	33 (30.60%)		
Hepatitis history	No	25 (27.80%)	28 (25.70%)	32 (29.60%)	0.421^a^	0.810
	Yes	65 (72.20%)	81 (74.30%)	76 (70.40%)		
Other chronic diseases	No	63 (70.00%)	39 (35.80%)	48 (44.40%)	24.403^a^	<0.001
	Yes	27 (30.00%)	70 (64.20%)	60 (55.60%)		
Treatment Modality	Surgical treatment	26 (28.90%)	37 (33.90%)	30 (27.80%)	5.945^b^	0.429
	Local therapy	11 (12.20%)	19 (17.40%)	14 (13.00%)		
	Systemic therapy	7 (7.80%)	5 (4.60%)	3 (2.80%)		
	Combined therapy	46 (51.10%)	48 (44.00%)	61 (56.50%)		
Disease knowledge	Knowledgeable	80 (88.90%)	90 (82.60%)	91 (84.30%)	1.621^a^	0.445
	Not knowledgeable	10 (11.10%)	19 (17.40%)	17 (15.70%)		

^a^ represents the χ^2^ value

^b^ represents Fisher’s exact test.; Class 1 (low fear-psychologically well-adapted group); Class 2 (high fear-social function concerns group); Class 3 (moderate-to-high fear-treatment concerns group)

Normality of the data was tested using skewness and kurtosis. The results showed that for the scores on the Fear of Cancer Recurrence, Perceived Social Support, Self-Efficacy, and Post-Traumatic Growth scales, the absolute values of skewness ranged from 0.261 to 0.544, and the absolute values of kurtosis ranged from 0.140 to 1.598, all meeting the criteria of absolute skewness < 1 and absolute kurtosis < 3. Each variable was approximately normally distributed; therefore, data were presented as mean ± standard deviation.

### 3.2 Common method bias test

Harman’s single-factor test identified 15 factors with eigenvalues exceeding 1, with the first factor accounting for 25.17% of the variance. This suggests that the study is free from common method bias.

### 3.3 Latent profile analysis of FCR in patients with primary liver cancer

As shown in **Table 2**, the AIC, BIC, and aBIC values decreased monotonically as the number of latent classes increased from one to five. Entropy values were above 0.80 for all models. The BLRT was significant for all five models, whereas the LMR-LRT for the three-profile model was not significant. Because the BLRT is known to outperform the LMR-LRT in simulation studies (27), we prioritized the BLRT result. Additionally, theoretical underpinnings play a crucial guiding role in classification decisions: According to the common-sense model of self-regulation (37), individuals’ cognitive representations of disease threats are heterogeneous, and FCR is not a single continuous experience but may differentiate into distinct adaptation patterns. Existing research suggests that high levels of FCR can at least be distinguished into two qualitatively different core concerns: one is about social functioning and interpersonal relationships (such as fear of being a burden to others or fear of being alienated) (14, 38), and the other is about the treatment process and disease progression (such as fear of treatment side effects or fear of poor treatment outcomes) (39, 40). Based on this theoretical distinction, we believe that a binary classification solution is too coarse (lumping together two qualitatively different high-fear types), while a four-class or higher solution may be overly fragmented, reducing clinical utility. This three-class structure also aligns with recent LPA studies of FCR in other cancer populations (7, 41). Posterior classification probabilities ranged from 87.8% to 95.2%, further supporting the validity of the three-profile solution. Accordingly, the three-profile model was selected based on a combination of statistical fit, clinical interpretability, and parsimony.

**Table 2:** Fit Indices for latent profile analysis of fear of cancer recurrence in patients with primary liver cancer.

Indices	1-profile	2-profile	3-profile	4-profile	5-profile
Fit statistics					
K	24	37	50	63	76
LL	-5160.388	-4869.366	-4768.192	-4702.243	-4650.696
AIC	10368.775	9812.732	9636.382	9530.485	9453.393
BIC	10458.220	9950.625	9822.726	9765.276	9736.633
aBIC	10382.102	9833.278	9664.148	9565.468	9495.594
Entropy	1	0.887	0.800	0.837	0.829
BLRT		<0.001	<0.001	<0.001	<0.001
LMRT		<0.001	0.250	0.098	0.582
Group-sizes (%)					
C1	307 (100%)	95 (30.95%)	90 (29.32%)	90 (29.32%)	82 (26.71%)
C2		212 (69.05%)	109 (35.50%)	70 (22.80%)	57 (18.57%)
C3			108 (35.18%)	37 (12.05%)	36 (11.73%)
C4				110 (35.83%)	84 (27.36%)
C5					48 (15.64%)

**Figure 2:**
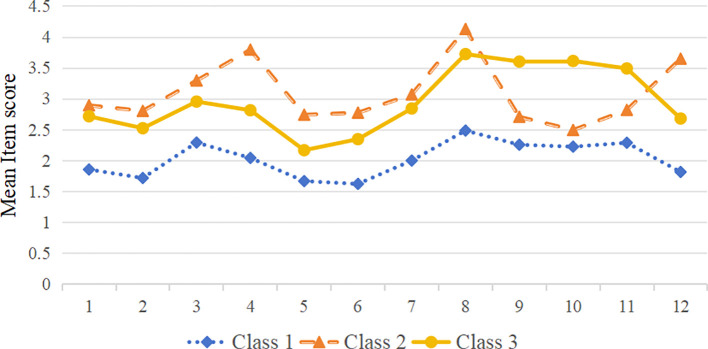
Latent profile characteristics of fear of cancer recurrence in patients with primary liver cancer. Class 1 (low fear–psychologically well-adapted group); Class 2 (high fear–social functioning concerns group); Class 3 (moderate-to-high fear–treatment concerns group).

Each profile was named based on differences in scores across various FCR items. The first profile consisted of patients with mild FCR and demonstrated a relatively good state of psychological adaptation; therefore, it was named the “low fear–psychologically well-adapted group” (29.32%). The second profile consisted of patients with the highest overall level of fear, with particularly high scores on items in the family/social dimension: Item 4 (“reduced work efficiency”), Item 8 (“being unable to continue hobbies due to illness”), and Item 12 (“thoughts of being unable to work”). These patients showed extreme concern about the loss of social roles and functions caused by the disease. Therefore, it was named the “high fear–social functioning concerns group” (35.50%). The third profile consisted of patients with a moderate-to-high overall level of fear. On the physical health dimension, the fear levels for Item 9 (“worry about major treatment”) and Item 10 (“worry about medication harming the body”) were much higher than those in the other two profiles, indicating that fear was highly concentrated on treatment-related concerns. Therefore, it was named the “moderate-to-high fear–treatment concerns group” (35.18%). As shown in **Figure 2**.

### 3.4 Comparison of PTG, perceived social support, and self-efficacy scores across various latent profiles in patients with liver cancer patients

As shown in **Table 3**, the latent profiles of FCR in patients with primary liver cancer differed significantly in terms of posttraumatic growth, perceived social support, and self-efficacy. Both total scores and dimension scores for these measures reached statistical significance (p < 0.05).

**Table 3:** Univariate analysis of scales across latent profiles of FCR (N = 307).

Variables	C1	C2	C3	F	P	Bonferroni
PTG	65.08 ± 9.05	55.5 ± 9.37	57.03 ± 11.13	25.67	<0.001	C1>C2, C1>C3
Relating to others	23.00 ± 4.21	21.71 ± 4.18	21.60 ± 4.57	3.093	0.047	C1>C3, C2>C3
New possibilities	9.54 ± 2.77	6.11 ± 2.33	7.25 ± 2.59	45.388	<0.001	C1>C3>C2
Personal strength	9.18 ± 2.29	7.33 ± 2.52	7.58 ± 2.85	14.438	<0.001	C1>C2, C1>C3
Spiritual change	10.48 ± 2.88	7.27 ± 2.96	8.03 ± 3.18	29.794	<0.001	C1>C2, C1>C3
Appreciation of life	12.88 ± 2.81	13.09 ± 2.98	12.56 ± 3.3	0.817	0.443	——
Perceived Social Support	64.81 ± 5.81	56.04 ± 8.86	55.21 ± 9.87	37.507	<0.001	C1>C2, C1>C3
Family support	25.03 ± 2.40	17.76 ± 4.41	19.06 ± 4.94	84.019	<0.001	C1>C3>C2
Friend support	17.10 ± 3.62	15.30 ± 4.16	14.50 ± 4.25	10.462	<0.001	C1>C2, C1>C3
Other support	22.68 ± 3.03	22.97 ± 2.83	21.65 ± 4.16	4.457	0.012	C1>C3, C2>C3
Self-efficacy	95.81 ± 13.76	76.62 ± 16.17	78.79 ± 18.38	39.535	<0.001	C1>C2, C1>C3
Positive attitude	49.79 ± 8.42	40.32 ± 8.60	40.78 ± 10.07	32.896	<0.001	C1>C2, C1>C3
Self-determination	10.79 ± 2.48	8.16 ± 2.64	8.52 ± 2.57	29.529	<0.001	C1>C2, C1>C3
Self-stress reduction	35.23 ± 5.17	28.15 ± 6.63	29.49 ± 7.30	32.205	<0.001	C1>C2, C1>C3

Class 1 (low fear-psychologically well-adapted group); Class 2 (high fear-social function concerns group); Class 3 (moderate-to-high fear-treatment concerns group)

### 3.5 Multinomial logistic regression analysis of factors associated with FCR profiles

Collinearity diagnostics showed that the variance inflation factor (VIF) for each variable ranged from 1.094 to 1.669, all below 5, and tolerance values ranged from 0.599 to 0.914, all above 0.1, indicating no severe multicollinearity among the independent variables.

The three latent classes of fear of cancer recurrence in patients with primary liver cancer were used as the dependent variable, with the “low fear–psychologically well-adapted” group set as the reference group. Variables that were statistically significant in the univariate analyses were entered as independent variables, with the following coding (1): employment status: 1 = in-service/employed, 2 = unemployed (2); residence: 1 = urban, 2 = rural (3); average monthly household income per capita: < 6,000 = 1, ≥ 6,000 = 2 (4); type of medical insurance: non-employee medical insurance = 1, employee medical insurance = 2 (5); presence of other comorbidities: absent = 1, present = 2. Perceived social support, self-efficacy, and post-traumatic growth were entered as measured values.

The results showed that type of medical insurance, presence of other comorbidities, employment status, perceived social support, self-efficacy, and post-traumatic growth were significant factors influencing different latent classes of fear of cancer recurrence in patients with primary liver cancer (p < 0.05), as shown in **Supplementary Table 1**.

### 3.6 Correlation analysis of FCR, perceived social support, self-efficacy and PTG

Significant intercorrelations emerged among key psychological variables in patients with liver cancer. These variables—FCR, perceived social support, self-efficacy, and PTG—are displayed in **Table 4**. FCR was negatively correlated with perceived social support, self-efficacy, and PTG (all p < 0.001). Perceived social support showed a positive correlation with self-efficacy and PTG (both p < 0.001). Self-efficacy showed a positive correlation with PTG (p < 0.001).

**Table 4:** The level and association of FCR, perceived social support, self-efficacy and PTG.

Variables	Mean	SD	1	2	3	4
1.FCR	32.81	6.70	1			
2.Perceived social support	58.32	9.45	-0.521^***^	1		
3.Self-efficacy	83.01	18.29	-0.483^***^	0.430^***^	1	
4.PTG	58.85	10.71	-0.472^***^	0.590^***^	0.452^***^	1

SD, Standard Deviation. ***P < 0.001.

### 3.7 Mediation analysis of perceived social support and self-efficacy between different FCR latent profiles and PTG

This study examined the chain mediating effect involving multi-category variables. First, the different profiles of FCR were treated as the independent variable. Post-traumatic growth was designated as the dependent variable. Perceived social support and self-efficacy were incorporated as mediating variables. A chain mediating effect test was then conducted to analyze these relationships. The latent profiles of fear of cancer recurrence were dummy coded, resulting in two dummy variables (X_1_ = “low fear–psychologically well-adapted group” vs. “high fear–social functioning concerns group”; X_2_ = “low fear–psychologically well-adapted group” vs. “moderate-to-high fear–treatment concerns group”). Additionally, mediation analyses were performed with type of medical insurance, presence of other comorbidities, and employment status included as control variables.

**Figure 3:**
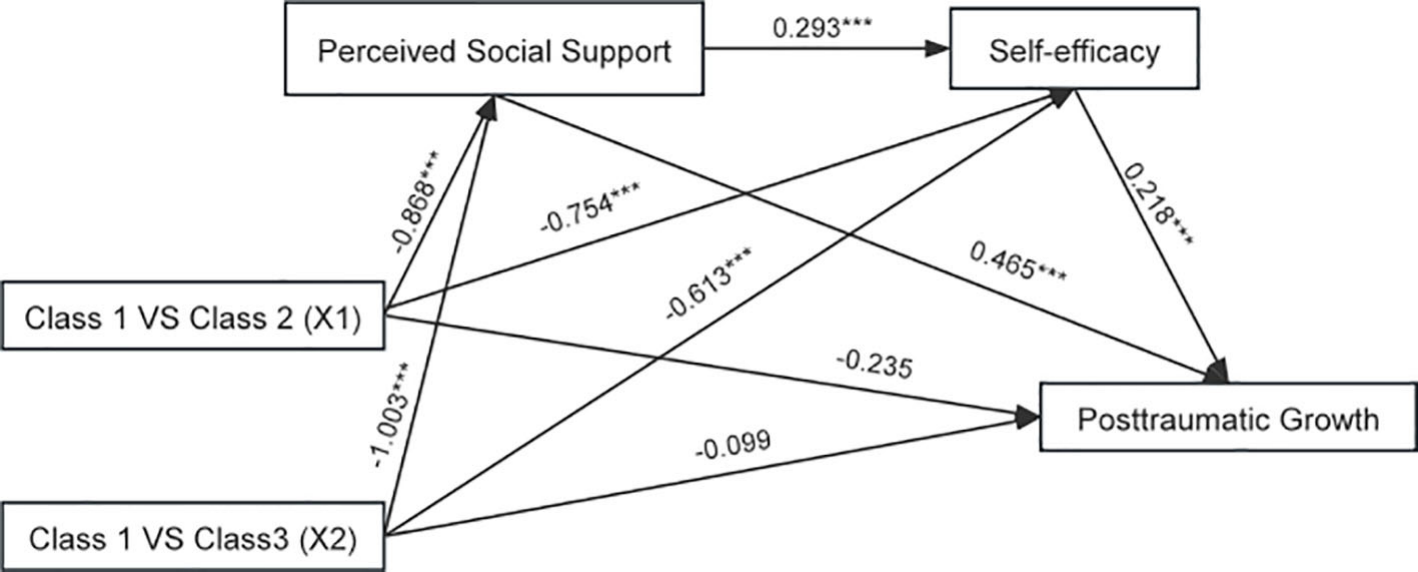
Relative serial mediation model of perceived social support and self-efficacy between different latent profiles of fear of cancer recurrence and posttraumatic growth. The low fear–psychologically well-adapted group was used as the reference group; X_1_ = “low fear–psychologically well-adapted group” vs. “high fear–social functioning concerns group”; X_2_ = “low fear–psychologically well-adapted group” vs. “moderate-to-high fear–treatment concerns group”. ***P < 0.001.

The results showed that the overall effect test was significant (F = 25.670, p < 0.001), indicating that different latent profiles of FCR differed significantly in their associations with PTG. Analysis conducted with the bootstrap method revealed that bootstrap 95% CIs for the relative mediation effects did not include zero, indicating that the relative mediation effects for both comparison groups were significant. Specifically, compared to the “low fear–psychologically well-adapted group”, the other two latent profiles exerted differential effects on PTG through the independent mediating roles of perceived social support and self-efficacy, as well as their sequential mediation. As shown in **Table 5**, **Figure 3**, and **Supplementary Table s2**.

**Table 5:** Relative mediation effect sizes across different latent profiles of fear of cancer recurrence.

Profiles	Mediation pathway	Effect	BootSE	BootLLCI	BootULCI	Effect proportion
X_1_	Relative total effect	-0.857	0.147	-1.147	-0.568	
	Relative direct effect	-0.235	0.136	-0.503	0.034	27.42%
	X_1_→Perceived social support→PTG	-0.403	0.086	-0.586	-0.251	47.02%
	X_1_→Self-efficacy→PTG	-0.164	0.051	-0.275	-0.072	19.14%
	X_1_→Perceived social support→Self-efficacy→PTG	-0.055	0.022	-0.105	-0.020	6.42%
X_2_	Relative total effect	-0.763	0.138	-1.034	-0.492	
	Relative direct effect	-0.099	0.130	-0.355	0.156	12.98%
	X_2_→Perceived social support→PTG	-0.466	0.086	-0.644	-0.303	61.08%
	X_2_→Self-efficacy→PTG	-0.133	0.046	-0.233	-0.054	17.43%
	X_2_→Perceived social support→Self-efficacy→PTG	-0.064	0.024	-0.118	-0.024	8.39%

The low fear–psychologically well-adapted group was used as the reference group; X_1_ = “low fear–psychologically well-adapted group” vs. “high fear–social functioning concerns group”; X_2_ = “low fear–psychologically well-adapted group” vs. “moderate-to-high fear–treatment concerns group”.

## 4 Discussion

This study employed latent profile analysis to reveal the heterogeneity of fear of cancer recurrence among patients with primary liver cancer and explored the path relationship between different FCR profiles and post-traumatic growth. Three distinct FCR latent profiles were identified, and it was revealed that perceived social support and self-efficacy played mediating roles between FCR profiles and post-traumatic growth. These findings provide a more detailed perspective for understanding the psychological adaptation process of this vulnerable group, distinguishing from the traditional understanding that simply views the relationship between FCR and post-traumatic growth as linear.

This study identified three FCR profiles, namely the “low fear–psychologically well-adapted group” (29.3%), the “high fear–social functioning concerns group” (35.5%), and the “moderate-to-high fear–treatment concerns group” (35.2%). This finding is similar to the results of Bentley et al. (7) and validates H1. The identification of these profiles indicates that FCR is not a single concept (23); in other words, patients with similar overall fear levels may have significantly different natures of fear. Although both the “high fear–social functioning concerns group” and the “moderate-to-high fear–treatment concerns group” reported high levels of fear of cancer recurrence, the content of their fears focused on different dimensions and items of the FCR scale. Specifically, the former scored highest on items related to work and hobbies (such as Item 4, “Reduced work efficiency due to illness,” and Item 12, “Worried about being unable to work”), reflecting deep concerns about the loss of social roles; the latter showed more prominent concerns on items related to treatment and side effects of medication (such as Item 9, “Worries about major treatment measures,” and Item 10, “Worried about medication harming the body”). This difference is consistent with the conservation of resources theory (42). This theory suggests that when individuals face a threat of loss, they strive to protect the resources they value. Therefore, the core threat for the “social functioning concerns group” lies in the potential loss of social and occupational resources, while for the “treatment concerns group”, the core threat stems more from the consumption of physical and energy resources required by treatment. Based on robust statistical fit indices and high posterior classification probabilities, cross-validation of the three-profile structure further supports the notion that, in the assessment of fear of cancer recurrence, it is not only necessary to focus on its severity but also to pay attention to the specific content of the fear tendency.

Multivariate logistic regression analysis further verified the characteristics of these profiles, revealing the influence of key sociodemographic and disease-related features. Notably, patients who were unemployed and not covered by employee medical insurance were more likely to belong to the high-fear group. This finding reinforces the resource-based explanatory logic—that is, unemployment implies the loss of important social roles and leads to an intensification of economic toxicity (43), which is particularly prominent for the “social functioning concerns group”, who are concerned about becoming a burden on their families or experiencing a decline in productivity (44). Similarly, the coexistence of other chronic diseases was associated with classification into the “high fear–social functioning concerns group” and the “moderate-to-high fear–treatment concerns group”. This might be due to the fact that the management of multiple chronic diseases adds pressure on personal and social resources, amplifying patients’ fears of functional decline and dependence on others (45). Moreover, the long-term treatment of multiple chronic diseases further exacerbates patients’ concerns about disease treatment (46).

The second hypothesis (H2) of this study was verified, indicating a significant association among fear of cancer recurrence, perceived social support, self-efficacy, and post-traumatic growth in patients with primary liver cancer. Fear of cancer recurrence was negatively correlated with post-traumatic growth (11). This finding supports the view that persistent and high levels of FCR can transform into a chronic stressor that hinders PTG, and it also aligns with the theory of life crisis and personal growth. According to this theoretical framework, the prerequisite for achieving post-traumatic growth through cognitive processing of traumatic events is the availability of sufficient psychological resources (12). High levels of fear of cancer recurrence that are not effectively managed will continuously deplete patients’ cognitive and emotional resources, leading to an increase in rumination and a depletion of coping abilities, ultimately hindering an individual’s post-traumatic growth (47, 48).

The third hypothesis (H3) of this study was also verified. This hypothesis focused on the mediating role of perceived social support and self-efficacy, thereby providing a preliminary process-oriented explanation for understanding the relationship between the fear of cancer recurrence profile and post-traumatic growth. The research results showed that the level of post-traumatic growth was lower in the other two groups compared to the low fear–psychologically well-adapted group, and the chain mediating effect for both groups was significant, indicating that perceived social support and self-efficacy played mediating roles in the relationship between different fear of cancer recurrence profiles and post-traumatic growth. This result is consistent with the core view of the theory of life crisis and personal growth—that is, persistent psychological stress will consume the internal resources needed for cognitive processing, thereby hindering the occurrence of post-traumatic growth (11). At the same time, a high degree of vigilance toward physical symptoms and a tendency to avoid social interaction may weaken the patient’s ability to perceive and utilize social support (49);while a strong sense of uncertainty about the future may also impair cancer-related self-efficacy—that is, individuals’ confidence in managing the disease (50). The chain mediating path suggests that there may be a cumulative effect of resource depletion. In other words, the decline in perceived social support further weakens belief in self-efficacy, thereby jointly constituting an obstacle to achieving post-traumatic growth.

It is worth noting that a refined comparison of the chain mediation paths based on the FCR profile reveals that, although both mediation paths are significant, the proportion of the effect of the moderate-to-high fear–treatment concerns group on post-traumatic growth through perceived social support (61%) is higher than that of the high fear–social functioning concerns group (47%). This suggests that, for the moderate-to-high fear–treatment concerns group, enhancing their perception of social support may be an important intervention point to promote post-traumatic growth (15). Treatment-related fears often involve specific and practical challenges, and emotional and instrumental support from the support network can directly buffer such distress (51). In contrast, for patients who are concerned about the loss of social function, their growth obstacles are more deeply intertwined with the weakening of personal initiative and self-worth, thus requiring a two-way intervention strategy to simultaneously enhance their perceived social support and self-efficacy.

Based on the above research findings, the results suggest that universal strategies for managing fear of cancer recurrence and promoting post-traumatic growth may not be effective (24). If intervention measures can be matched with the FCR profiles of patients with primary liver cancer, the intervention effect may be improved. For patients in the moderate-to-high fear–treatment concerns group, intervention measures that clearly activate and strengthen their perception of available support—such as couple-based dyadic coping and family empowerment—can enhance family care, directly alleviate treatment-related anxiety, and motivate patients to achieve positive psychological growth. For the high fear–social functioning concerns group, a more integrated intervention approach is needed (52). In addition to enhancing social support, the intervention should focus on rebuilding self-efficacy. Activities such as gratitude journals and peer support groups can help patients transform positive feedback and coping experiences into beliefs in self-efficacy, thereby reducing fears of losing social roles and restoring a sense of competence, ultimately facilitating post-traumatic growth (53, 54). Moreover, for both high fear–social functioning concerns and moderate-to-high fear–treatment concerns groups, the impact of economic toxicity should be considered, and appropriate social work referral services should be provided to address possible structural barriers that may exacerbate fear of cancer recurrence (44, 55).

In conclusion, this study has demonstrated that fear of cancer recurrence is not a single homogeneous concept. Its different facets predict post-traumatic growth levels differently by weakening perceived social support and self-efficacy, thereby further dissecting the complex relationship between fear of cancer recurrence and post-traumatic growth. By identifying subtypes of fear of cancer recurrence that have both theoretical and clinical significance, this study highlights the importance of precise intervention in psychosocial care for cancer patients. Future intervention practices should consider focusing on the specific categories of patients’ fears and their corresponding resource deficiencies, thereby more effectively creating conditions for liver cancer patients to realize their potential for post-traumatic growth after experiencing the disease.

### 4.1 Limitations

This study has several limitations. First, the cross-sectional design precludes any possibility of making causal inferences. The observed relationships are all associative, and their causal effects need to be verified by longitudinal studies. Second, the study relied entirely on self-report measurement tools, which are susceptible to recall bias and social desirability bias. Future studies could consider incorporating clinical physician evaluations or behavioral measurement indicators. Third, the sample of this study was from a single country and mainly consisted of male patients with primary liver cancer, which may limit the generalizability of the study results to other cultural backgrounds or cancer types with different gender compositions. Finally, although latent profile analysis identified three theoretically coherent profiles, future studies with larger and more diverse samples are needed to confirm the stability and reproducibility of these profiles.

## 5 Conclusion

This cross-sectional exploratory study identified three latent profiles of FCR in patients with primary liver cancer and found that perceived social support and self-efficacy mediated the relationship between different latent profiles of FCR and PTG. Based on these findings, clinical practice may attend to the heterogeneous characteristics of FCR among patients and explore corresponding intervention strategies according to different fear subtypes. By integrating social support resources and enhancing patients’ self-efficacy, it may be possible to facilitate post-traumatic growth in patients with primary liver cancer.

## Data Availability

The original contributions presented in the study are included in the article/**Supplementary Material**. Further inquiries can be directed to the corresponding authors.
