# Supplementary material for: Associations between fear of cancer recurrence and post-traumatic growth in patients with primary liver cancer: a latent profile analysis and mediation analysis
Source: Front Psychiatry. 2026 Jun 8;17:1819344. doi: 10.3389/fpsyt.2026.1819344 (PMC13284152; doi:10.3389/fpsyt.2026.1819344)
Supplement: Supplementary file 1 [file Table1.docx]

**Supplementary Table S1** Multivariable Regression Analysis of Latent Profiles of Fear of Cancer Recurrence in Patients With Primary Liver Cancer

| Variables | *β* | *SE* | *Wald χ^2^* | *P* | *OR* | 95% *CI* |
| --- | --- | --- | --- | --- | --- | --- |
| C2 VS C1 | | | | | | |
| Type of health insurance (employee medical insurance as the reference) | | | | | | |
| Non-employee medical insurance | 1.875 | 0.435 | 18.605 | ＜0.001 | 6.518 | 2.781~15.277 |
| Other chronic diseases (yes as the reference) | | | | | | |
| No | -1.808 | 0.408 | 19.602 | ＜0.001 | 0.164 | 0.074~0.365 |
| Occupation status (not employed as the reference) | | | | | | |
| In-service/Employed | -0.091 | 0.405 | 0.050 | 0.823 | 0.913 | 0.413~2.020 |
| Perceived social support | -0.088 | 0.029 | 9.325 | 0.002 | 0.916 | 0.866~0.969 |
| Self-efficacy | -0.063 | 0.014 | 21.685 | ＜0.001 | 0.939 | 0.914~0.964 |
| PTG | -0.054 | 0.024 | 5.175 | 0.023 | 0.947 | 0.904~0.992 |
| C3 VS C1 | | | | | | |
| Type of health insurance (employee medical insurance as the reference) | | | | | | |
| Non-employee medical insurance | 0.793 | 0.422 | 3.522 | 0.061 | 2.209 | 0.965~5.056 |
| Other chronic diseases (yes as the reference) | | | | | | |
| No | -0.984 | 0.388 | 6.439 | 0.011 | 0.374 | 0.175~0.799 |
| Occupation status (not employed as the reference) | | | | | | |
| In-service/Employed | -0.879 | 0.403 | 4.758 | 0.029 | 0.415 | 0.188~0.915 |
| Perceived social support | -0.131 | 0.028 | 21.211 | ＜0.001 | 0.878 | 0.830~0.928 |
| Self-efficacy | -0.057 | 0.013 | 19.452 | ＜0.001 | 0.944 | 0.921~0.969 |
| PTG | -0.029 | 0.023 | 1.672 | 0.196 | 0.971 | 0.929~1.015 |

Note:Class 1 (low fear–psychologically well-adapted group); Class 2 (high fear–social function concerns group); Class 3 (moderate-to-high fear–treatment concerns group).

**Supplementary Table S2** Testing the Relative Chain Mediation Effects of Fear of Cancer Recurrence on Post-traumatic Growth

| Variables | Model 1  (PTG） | | Model 2（Perceived social support） | | Model 3（Self-efficacy） | | Model 4  （PTG） | |
| --- | --- | --- | --- | --- | --- | --- | --- | --- |
|  | *β* | *t* | *β* | *t* | *β* | *t* | *β* | *t* |
| Occupation status | 0.092 | 0.795 | 0.007 | 0.061 | 0.057 | 0.533 | 0.076 | 0.784 |
| Type of health insurance | 0.133 | 1.157 | 0.163 | 1.467 | -0.056 | -0.530 | 0.059 | 0.610 |
| Other chronic diseases | 0.016 | 0.138 | -0.001 | -0.006 | -0.146 | -1.366 | 0.048 | 0.494 |
| X_1_ | -0.857 | -5.838^***^ | -0.868 | -6.102^***^ | -0.754 | -5.246^***^ | -0.235 | -1.721 |
| X_2_ | -0.763 | -5.544^***^ | -1.003 | -7.527^***^ | -0.613 | -4.426^***^ | -0.099 | -0.766 |
| Perceived pocial support |  | |  | | 0.293 | 5.332^***^ | 0.465 | 8.900^***^ |
| Self-efficacy |  | |  | |  | | 0.218 | 4.153^***^ |
| *R^2^* | 0.151 | | 0.204 | | 0.280 | | 0.407 | |
| *F* | 10.704^***^ | | 15.418^***^ | | 19.410^***^ | | 29.370^***^ | |

Note：^***^*P*＜0.001; The low fear–psychologically well-adapted group was used as the reference group; X₁ = “low fear–psychologically well-adapted group” vs. “high fear–social functioning concerns group”; X₂ = “low fear–psychologically well-adapted group” vs. “moderate-to-high fear–treatment concerns group”.
